# A 2.4 GHz Wide-Range CMOS Current-Mode Class-D PA with HD2 Suppression for Internet of Things Applications

**DOI:** 10.3390/s24051616

**Published:** 2024-03-01

**Authors:** Nam-Seog Kim

**Affiliations:** Department of Information and Communication Engineering, School of Electrical and Computer Engineering, Chungbuk National University, Cheongju-Si 28644, Republic of Korea; namseog.kim@cbnu.ac.kr

**Keywords:** bluetooth low energy (BLE), class-D power amplifier (PA), complementary metal–oxide–semiconductor (CMOS), current-mode, second harmonic distortion (HD2), internet of things (IoT), transmitter (TX), power efficiency, wireless sensor network (WSN), Zigbee

## Abstract

Short-range Internet of Things (IoT) sensor nodes operating at 2.4 GHz must provide ubiquitous wireless sensor networks (WSNs) with energy-efficient, wide-range output power (POUT). They must also be fully integrated on a single chip for wireless body area networks (WBANs) and wireless personal area networks (WPANs) using low-power Bluetooth (BLE) and Zigbee standards. The proposed fully integrated transmitter (TX) utilizes a digitally controllable current-mode class-D (CMCD) power amplifier (PA) with a second harmonic distortion (HD2) suppression to reduce VCO pulling in an integrated system while meeting harmonic limit regulations. The CMCD PA is divided into 7-bit slices that can be reconfigured between differential and single-ended topologies. Duty cycle distortion compensation is performed for HD2 suppression, and an HD2 rejection filter and a modified C-L-C low-pass filter (LPF) reduce HD2 further. Implemented in a 28 nm CMOS process, the TX achieves a wide P_OUT_ range of from 12.1 to −31 dBm and provides a maximum efficiency of 39.8% while consuming 41.1 mW at 12.1 dBm P_OUT_. The calibrated HD2 level is −82.2 dBc at 9.93 dBm P_OUT_, resulting in a transmitter figure of merit (TX_FoM) of −97.52 dB. Higher-order harmonic levels remain below −41.2 dBm even at 12.1 dBm P_OUT_, meeting regulatory requirements.

## 1. Introduction

A wireless sensor network (WSN) consists of a large number of infrastructure-less wireless sensors deployed in an ad hoc manner, where each sensor node is used to monitor system, physical, or environmental conditions in a specific area [1]. The nodes in any WSN contain sensor interfaces, computing devices, transceivers, and power devices. These devices perform important tasks by enabling the nodes to communicate with each other and transmit data obtained from sensors. The need for this system has led to the development of the concept of the Internet of Things (IoT). The IoT allows for instant access to environmental data, which greatly increases efficiency and productivity in many processes [2].

**Figure 1:**
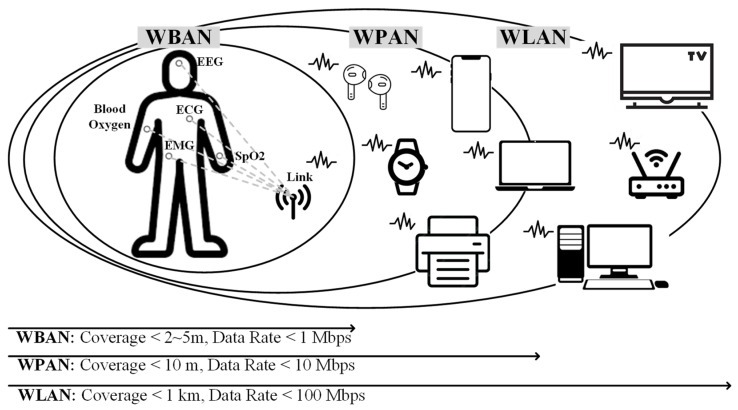
WBAN and WPAN for wireless sensor networks.

The sensor nodes support both wireless personal area networks (WPANs) for long-range applications, such as smart homes [3], and wireless body area networks (WBANs) for short-range applications, such as low-data medical monitoring [4] as shown in Figure 1. Bluetooth Low Energy (BLE) or Zigbee using long-range energy-efficient wireless systems [5,6,7,8] are the most widely used for WPANs. Most wireless transmitters (TXs) are optimized to be most efficient at output powers above 10 dBm for WPAN applications to achieve high efficiency. Thus, they are less efficient in applications using output powers as low as −20 dBm with the same IoT sensors. As IoT sensor nodes become more ubiquitous, the sensor node systems need to support both WPANs and WBANs with high efficiency to maximize battery life. Ubiquitous networking for IoT aims to provide seamless communication between humans and things, and between humans and things while moving from one place to another. With the help of ubiquitous networking, things can recognize their characteristics, context, and situations. Smart objects use the Internet as a communication infrastructure to share and process information such as their identity, current location, physical characteristics, and information they perceive around them.

The most vital and energy-hungry part is the power amplifier (PA), which consumes more than 50% of the available power in the IoT sensor node [9]. This serves as the major cause of low battery life in any wireless transceiver. Switched-mode PA architectures are commonly used for IoT applications since the phase-modulated signals used in such systems do not have any amplitude information. Popular architectures are class-D [10,11,12,13] and class-E/F PAs [14,15]. Class-E/F PAs usually use cascading for reliability concerns and more complicated off-chip matching networks for harmonic suppression [16]. Class-D PAs are more suitable for applications targeting output powers around 10 dBm with robust matching and no reliability concerns. Therefore, the output power control across a wide range of >30 dB remains a challenge.

**Figure 2:**
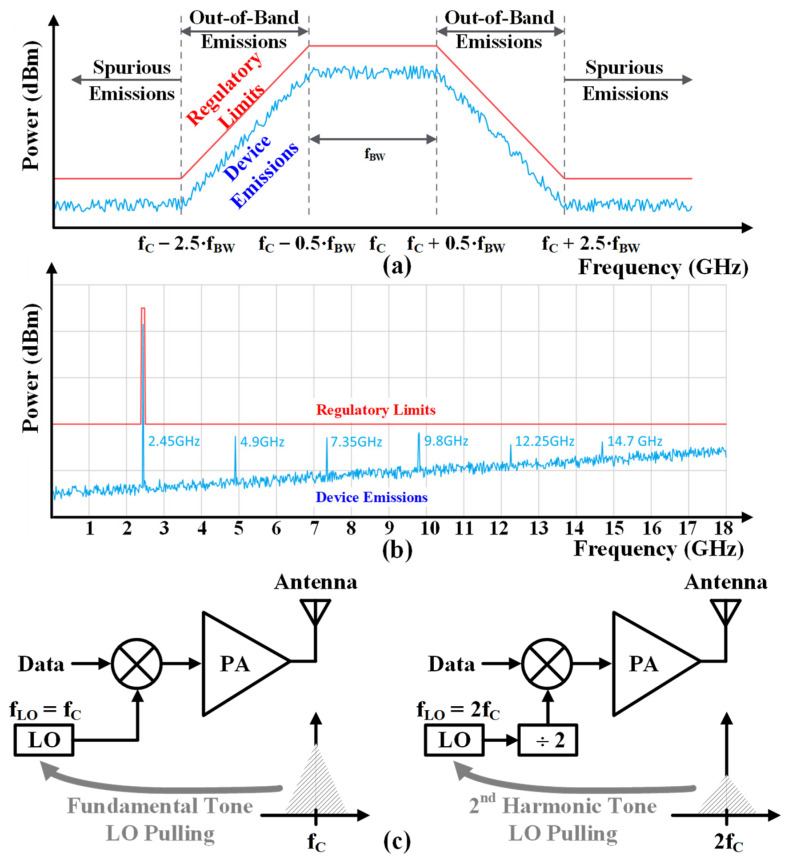
(**a**) electromagnetic compatibility and (**b**) harmonic spurious emission regulation of BLE and (**c**) oscillator pulling due to high power outputs of the PA.

PAs also generate electromagnetic waves at unintended frequencies. Strong harmonic spurious emissions can potentially contaminate the out-of-band spectrum, causing receivers (RXs) operating at the same harmonic frequencies to desensitize. As a sensor node operating in the industrial, scientific, and medical (ISM) band, spurious emissions are categorized as any electromagnetic emissions that occur at frequencies that are not intentionally emitted, especially for electronics that intentionally emit one or more frequencies. Therefore, all electronics are required to be tested to ensure that they do not emit electromagnetic waves of excessive intensity at all frequencies except those at which they are intentionally emitted, which is known as electromagnetic compatibility (EMC) testing as shown in Figure 2a. Furthermore, the TX must meet more stringent harmonic levels corresponding to conducted power of <−41.2 dBm as shown in Figure 2b. For example, the second harmonic (HD2) of a 2.4 GHz TX is located at the N79 band of a 5 G NR [14]. In addition to the interference effect on devices operating at around 4.8 GHz, a high-power PA is a high self-interference source that leads injection pulling of the voltage-controlled oscillator (VCO) in a chip. When a PA is integrated on the same chip with a VCO, as shown in Figure 2c, if the PA output frequency and local oscillator (LO) frequency are the same, the high output of the fundamental frequency of the PA causes pulling for the LO. Even when the operating frequency of the PA is half that of the LO frequency, the second harmonic of the PA can cause the VCO pulling [17].

This paper introduces a TX with a digitally controllable current-mode Class-D (CMCD) PA, second-harmonic distortion (HD2) super-compression, and passive filters for harmonic component reduction. It employs digitally controlled multi-slice switches and dual-mode operation in differential and single-ended PA configurations to handle a wide range of output power for a variety of ubiquitous IoT applications. It also reduces HD2 distortion with duty-cycle compensation, an on-die HD2 rejection filter, and an externally modified CLC low-pass filter (LPF). This article is organized in the following manner. Section 2 presents an overview of class-D PAs. Section 3 describes the overall block diagram of the proposed TX and the wideband CMCD PA with harmonic suppression technique. Section 4 discusses the experimental results of the proposed TX obtained on the integrated circuit fabricated in 28 nm CMOS technology. Finally, the main contributions are summarized in Section 5.

## 2. Class-D Power Amplifier Overview

Switched-mode PAs have the characteristic that the product of the voltage and current waveforms applied to the transistor becomes zero, so the power consumed by the transistor becomes zero, showing a theoretical efficiency of 100%. However, the theoretical high efficiency cannot be obtained due to the effect of parasitic components present in the transistor, and the loss due to parasitic components increases as the operating frequency increases, which limits its use in high-frequency bands. Class-E/F PAs have received much attention as amplifiers for wireless power transmission systems due to their ease of use, heat dissipation, and simple structure. However, compared to other PAs, they are less efficient at utilizing high power and require switch elements with high breakdown voltages due to their high drain-source peak voltages [18]. Unlike Class-E/F PAs, the Class-D PAs have a lower output current as the load impedance increases. Therefore, the Class-D PAs have less efficiency change as the load impedance changes since the loss of the transistor is relatively reduced due to the reduced current.

**Figure 3:**
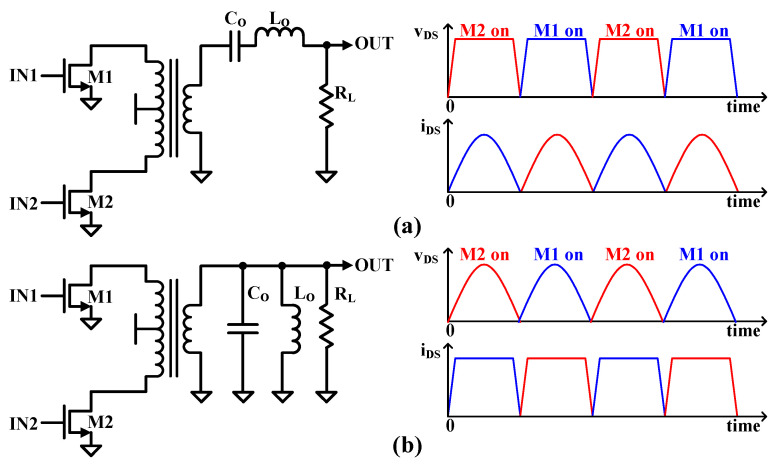
(**a**) Voltage mode class-D PA (VMCD) and (**b**) current mode class D PA (CMCD).

The class-D PAs are categorized as voltage-controlled and current-controlled. As shown in Figure 3a, a voltage-mode class-D (VMCD) PA operates two transistors by switching each transistor on and off with a 180° phase difference, which causes the drain voltage waveform to be a square wave, and only the desired frequency components are delivered to the load through a series resonant circuit connected to the load. The series filter has a resonant frequency that is set to the center frequency of the output signal. The voltage across the transistor is a square wave and the transistor current will be a half-wave rectified sine wave, which is theoretically 100% efficient. However, the efficiency of the transistor can be reduced if there is some parasitic drain-source capacitance (*C_DS_*) present in the transistor that must be charged, discharged, or grounded through the transistor. This means that the voltage waveform will not have a perfectly square shape, and some transient current spikes will occur when the transistor turns on. The overlap of voltage and current is unavoidable. Energy dissipation (*E*) is defined as Equation (1) during each transistor closure cycle.

(1) $$E=\frac{1}{2}C_{DS}V_{DD}^{2}$$

where *V_DD_* is supply voltage. The inevitable overlap of voltage and current in VMCD Pas, therefore, results in a significant loss of energy efficiency from an ideal class-D PA and limits the application of low-power IoT sensors in gigahertz operating applications. For the current-mode class-D (CMCD) PA as shown in Figure 3b, the two switching transistors control the current instead of the voltage. Moreover, a parallel filter is set to the center frequency of the output signal. The filter resonance causes zero-voltage switching, and there is no voltage across the transistor during each transistor closure cycle. There are filters connected in parallel and their resonance frequency is set to the carrier frequency—the filter resonance results in zero-voltage switching with no voltage across the transistor at each switching time. Even if the transistor has some output CDS, the CDS can be part of the output parallel filter. Thus, current-mode Class-D PAs have high-efficiency characteristics and very small parasitic losses for design PAs in the gigahertz range [19].

## 3. Wide-Range CMCD PA with Harmonic Suppression

### 3.1. Transmitter Architecture

**Figure 4:**
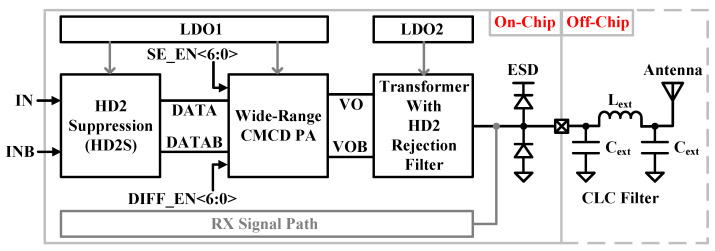
Proposed transmitter architecture block diagram.

Figure 4 shows the proposed TX architecture. Gaussian minimum shift keying (GMSK) based on two-point modulated data of a phase-locked loop is used for the differential inputs (IN/INB) of the TX. The HD2 suppression (HD2S) block is used to reduce HD2, which can mitigate VCO pulling and meet out-of-band spurious emissions, including second and third harmonic distortion (HD3) magnitudes below absolute −41 dBm. The following CMCD PA provides a wide range of output power with digital control signals of SE_EN<6:0> and DIFF_EN<6:0>. The CMCD PA is connected to a 3:2 transformer (XFMR) to provide impedance matching and a differential signal as a single-ended signal for driving an external antenna. The XFMR also includes an LC parallel HD2 rejection filter to further reject HD2. Two low-dropout voltage regulators are used to provide stable supply voltages. The first LDO output is assigned for thin oxide transistors that are used in all TX blocks except the CMCD PA block. The second LDO output is connected to the XFMR center tap to provide the power to the CMCD PA arrays using nMOSFET transistors and isolate other circuitries from high-power PA. Two low-dropout voltage regulators are used to provide a stable supply voltage, and their outputs are 1.0 V. A typical electrostatic discharge (ESD) protection circuit with double diodes is added at the PAD. An external CLC π-type low-pass filter is added right next to the external PAD to reduce harmonics additionally.

### 3.2. Wide-Range CMCD Power Amplifier

**Figure 5:**
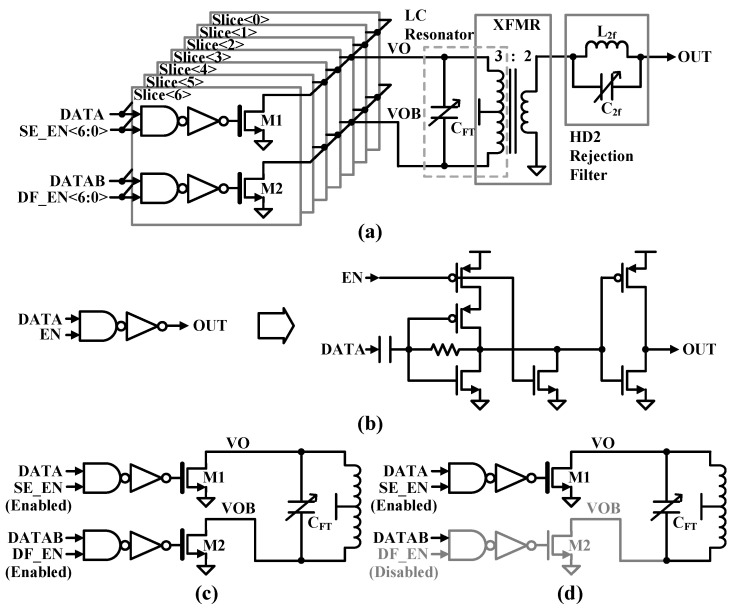
(**a**) Wide-range CMCD PA, (**b**) AND gate, and (**c**) differential and (**d**) single-ended CMCD.

Figure 5a shows a wide-range CMCD PA. The PA consists of two thick oxide nMOSFETs to withstand the high output power. The C_DS_ of M1 and M2 can be designed to be included in the capacitor of the LC resonator, which is a differential amplification load for the tuned amplifier. The primary inductor (L_FTP_) of the transformer (XFRM) serves as the inductance in the CMCD PA tuning load also. As shown in Figure 5b, an AND gate consisting of a NAND and an inverter drives the nMOSFETs in the CMCD PA and provides digital gain selection with the SE_EN<6:0> and DF_EN<6:0> signals. The resistive feedback increases the 3 dB bandwidth compared to a conventional static NAND gate. AC coupling completely blocks the common-mode level of the clock signal to prevent propagation of duty-cycle distortion. Self-biasing of the crossover voltage restores the duty cycle to its ideal value regardless of the input duty cycle. AC coupling combined with the low-pass characteristics of the inverter produces bandpass characteristics. The bandpass filter suppresses phase noise in the input data because it attenuates all out-of-band noise. The digital variable gain PA is organized into 7-bit binary slices with a LSB slice of 2.5 μm/150 nm transistor size to implement 127 differential output powers as shown in Figure 5c. The PA also supports a single-ended mode to further fine-tune the gain steps as shown in Figure 5d. The DF signal coming into the input is disabled to operate in single-ended mode within each slice, which forces one side of the path (the negative side) to be turned off, resulting in 6 dB lower output power compared to differential mode.

### 3.3. Transformer and HD2 Rejection Filter

**Figure 6:**
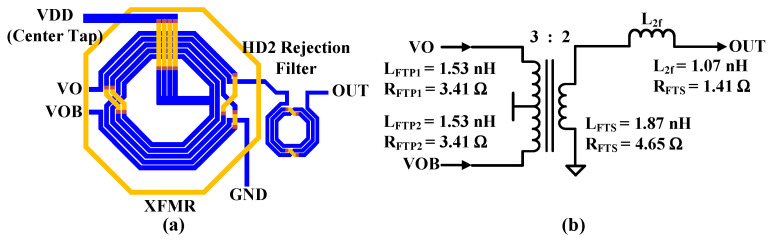
(**a**) XFMR and inductor of HD2 rejection filter and (**b**) their equivalent circuit.

Figure 6a shows the transformer (XFMR) and HD2 rejection filter inductor block used for the proposed CMCD PA. The XFMR implements the main inductor and connection crossings with two top metal lines while meeting the electrical migration rules. A ring structure is added around the XFRM to reduce unwanted second-harmonic coupling to the nearby circuits including the HD2 rejection filter inductor and the VCO inductor. A 3D electromagnetic simulation is performed to obtain the extracted values of the XFMR and inductor, and the corresponding schematics and their values are shown in Figure 6b. The larger the inductance, the more favorable it is for increasing the Q-factor of the resonant circuits. The switched capacitor (C_FT_) allows discrete frequency tuning for coverage of 2.4 GHz ISM band (2.4~2.48 GHz) resonating with parallel L_FTP_.

The HD2 rejection filter is implemented as an LC band-stop filter or notch filter as shown in Figure 5a. The parallel LC components of *L_2f_* and *C_2f_* exhibit high impedance at their resonant frequency, twice the 2.4 GHz ISM band, so they block the HD2 signal from the load at that frequency. Conversely, at other frequencies, they pass the signal to the load. The resonant frequency of the HD2 rejection filter is expressed by Equation (2).

(2) $$f_{HD2}=\frac{1}{2\pi \sqrt{L_{2f}C_{2f}}}$$

**Figure 7:**
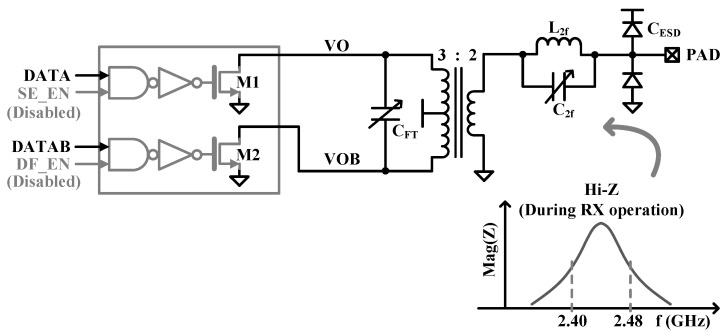
Embedded low-loss TX switch for RX operation mode.

The embedded low-loss switch scheme [14] is applied for the use of a single antenna to support time division duplex (TDD) operation. The switch of the proposed TX (TX_SW) has a high impedance of more than 300 Ωs within the 2.4 GHz ISM band in RX operation mode as shown in Figure 7. The XFMR multiplies the C_FT_ by the square of the primary to secondary-turn ratio (n^2^). All PA slices are disabled, and the n^2^ C_FT_ output capacitance by the XFMR plus the 50% ESD capacitance (*C_ESD_*) at the PAD due to the RX sharing model resonates with the secondary inductance (*L_FTS_*) of the XFMR to ensure that the TX has a high output impedance during RX operation. The CFT must support different capacitance values during TX and RX operations. A typical capacitance is about 1200 fF in TX operation mode and about 450 fF in RX operation mode. Therefore, the C_FT_ must accommodate a wide capacitance tuning range, which limits the increase in inductance value to increase the Q-factor of the XFMR, so half of the L_FTP_ in the proposed TX is set to about 1.5 nH. The resonance frequency of the *TX_SW* is given by Equation (3), and the frequency is tuned at around 2.44 GHz to get high impedance during RX operation mode.

(3) $$f_{TX\_SW}=\frac{1}{2\pi \sqrt{(0.5C_{ESD}+n^{2}C_{FT})L_{FTS}}}$$

An impedance matching is achieved by using the 3:2 XFMR (n = 2/3) and the HD2 rejection filter between differential high-output impedance of the PA and single-ended low impedance of an antenna in the TX mode. The XFMR changes impedance of CMCD PA by multiplying n^2^. The series LC band-stop filter tuned at HD2 frequency operates as mostly inductance at fundamental frequency and makes L matching network.

**Figure 8:**
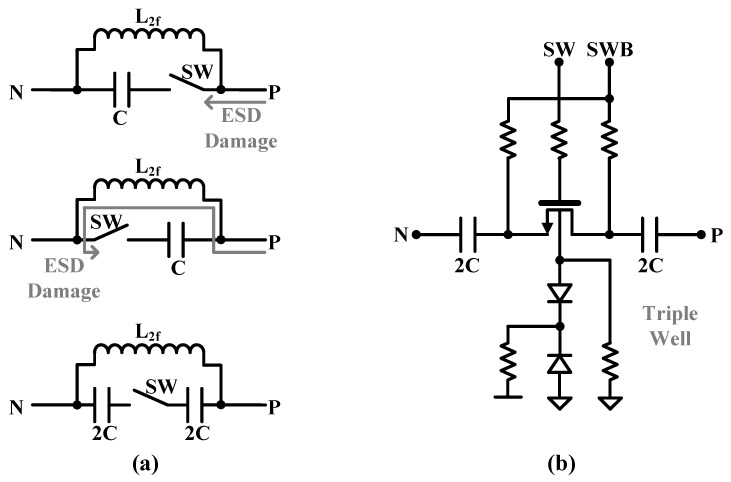
(**a**) Single-ended and differential switched-capacitor scheme for HD2 rejection filter and (**b**) differential switched-capacitor configuration using triple-well.

The proposed CMCD PA has two switched capacitors. One is the primary resonant capacitor of the XFMR, and the other is for the HD2 rejection filter. Since the CMCD PA is a differential circuit, the switched capacitors must also be differential, which uses two 2× capacitors in series compared to a single-ended structure. For the HD2 rejection filter, since it is a single-ended filter, a single-ended switch capacitor structure can be applied. However, a differential structure should also be used for the HD2 rejection filter because, in a single-ended structure, the switch thick oxide nMOSFETs are directly connected to the PAD, making them vulnerable to ESD damage as shown in Figure 8a. In the differential configuration, there is no direct current path from the PAD. The differential configuration uses the triple-well that helps float the device’s body voltage to prevent junction failures due to potentially large swings across all nodes of the switch as shown in Figure 8b. Similarly, the gate, drain, and source nodes are also floated while being DC-biased through large resistors. A large resistor of 20 kΩ is chosen to minimize the insertion loss of the switch. When the switch is on, the source and drain node DC voltages are pulled to GND. When the switch is off, the source and drain node DC voltages are pulled to VDD. This prevents the junction from being biased forward due to the swing seen on the other port after the switch is turned on, potentially degrading linearity or gain. The switch transistor size is 20 μm/150 nm.

### 3.4. HD2 Suppression with Duty-Cycle Correction

**Figure 9:**
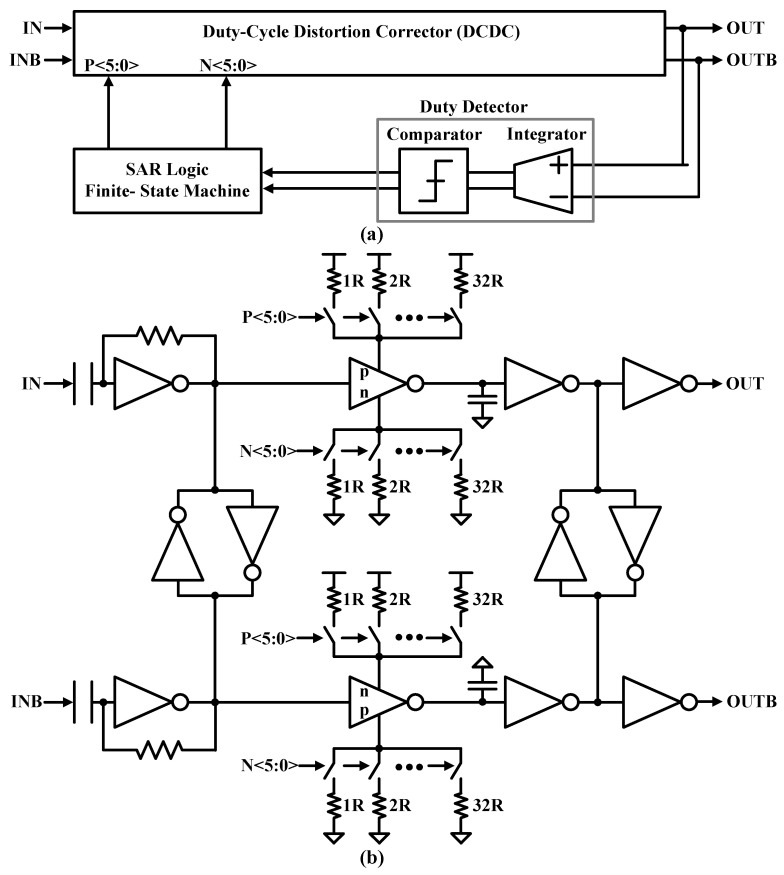
(**a**) HD2 suppression block diagram and (**b**) digitally controlled DCDC circuit.

Differential PA topologies are commonly used to suppress HD2 emissions, which can violate spurious emission limits in wireless standards. However, its effectiveness is limited by device mismatch and asymmetry. The HD2 is caused by the input data duty-cycle imbalance, so the proposed TX employs a duty-cycle correction circuit (DCC) before driving the CMCD PA. Figure 9a shows the block diagram of the successive approximation register (SAR)-DCC circuit, which consists of a duty-cycle distortion corrector (DCDC), a duty-cycle detector (DCD), and a SAR logic controller. The DCD essentially consists of an analog integrator and a comparator. The integrator is a fully differential charge pump with a common-mode feedback circuit, and the fully differential dynamic comparator is based on two cross-coupled differential pairs with switched current sources loaded with a CMOS latch. The output of the comparator is given to the SAR logic controller that adopts the binary search algorithm and adjusts load capacitances in the DCDC to correct the input duty cycle by 50%. The duty-cycle error correction loop is continuously running to compensate for voltage and temperature drift. A 10 MHz clock is used to run the error detection and correction logic. 

Figure 9b shows the digitally controlled DCDC circuit. The first stage of the DCDC, an AC-coupled resistive feedback inverter, compensates for the duty-cycle error to some extent on its own. This is done by storing the average value of the input in an AC capacitor. Change the common-mode voltage at the input of the first-stage inverter. The DCDC calibration is performed down to 1.5 ns with a resolution of up to ±45 ns in the time domain by the digital control words P<5:0> and N<5:0>, which independently adjusts the pull-up and pull-down resistors of the delay device. This corresponds to a correction operation of ±32 degrees in the phase domain, with a resolution of approximately 0.5 degrees at a data rate of 2 Mb/s.

### 3.5. External CLC Low-Pass-Filter for Harmonic Suppression

**Figure 10:**
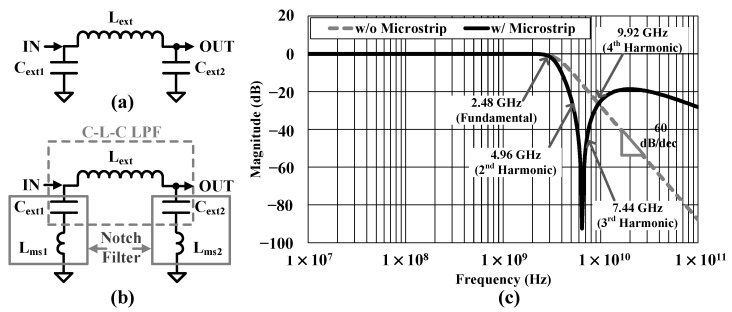
(**a**) C-L-C LPF, (**b**) modified C-L-C having microstrip inductance LPF, and (**c**) their magnitude frequency response.

An external C-L-C filter consisting of a capacitor-inductor-capacitor in a π configuration creates a low-pass filter (LPF) for harmonics suppression as shown in Figure 10a. The input capacitor (*C_ext1_*) effectively filters the CMCD PA switching harmonics and helps to reduce the capacity and size of each component. The *C_ext1_* provides very low reactance to the ripple frequency, so the main part of the filtering is done by C_ext1_. Most of the residual ripple is removed by the combined effect of *L_ext_* and *C_ext2_*. The C-L-C filter contains three L or C elements. Hence, the filter is essentially a third-order filter and provides −60 dB/decade of roll-off at frequencies above a cutoff frequency of $1/2\pi \sqrt{L_{ext}C_{ext}}$, where *C_ext_* is 1 pF, the same as *C_ext1_* and *C_ext2_*, and *L_ext_* is 4 nH. However, it only provides HD2 suppression of 9 dB as the second harmonic frequency is close to the fundamental frequency as shown in Figure 10c.

Microstrip inductance of 600 pH is added between *C_ext_* to GND path on a printed circuit board (PCB), which can add two notch filters in addition to C-L-C LPF as shown in Figure 10b. The resonance frequency of the notch filters is 6.5 GHz, which is between the second and third harmonic frequency, and it gives HD2 suppression of 23 dB and HD3 suppression of 42 dB. The suppression is much higher than the simple C-L-C LPF. They have the same HD4 suppression. Harmonic components greater than HD4 are lesser than the regulatory limit of −41.2 dBm without the external filter.

## 4. Experimental Results

**Figure 11:**
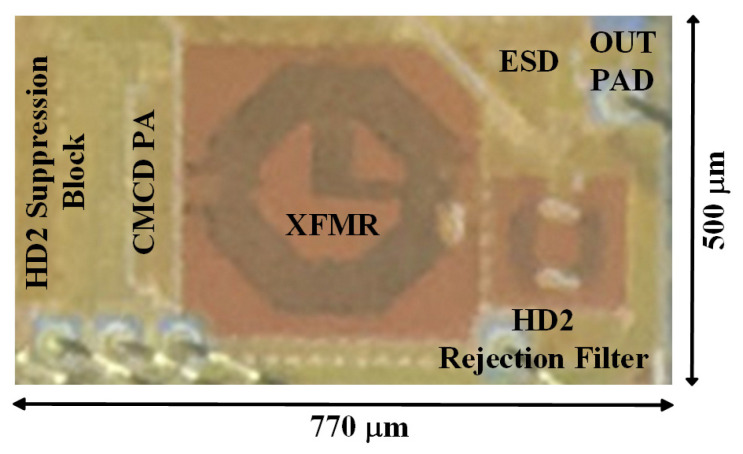
Microphotograph of the proposed transmitter.

Figure 11 shows a micrograph of the proposed TX implemented in a 1P8M 28-nm CMOS process. It includes the HD2 suppression block, the CMCD PA, the XFMR, the HD2 rejection filter, the ESD diodes on the die, and input/output PADs. The die area is 0.385 mm^2^. The C-L-C LPF is attached to the output PAD on the PCB.

**Figure 12:**
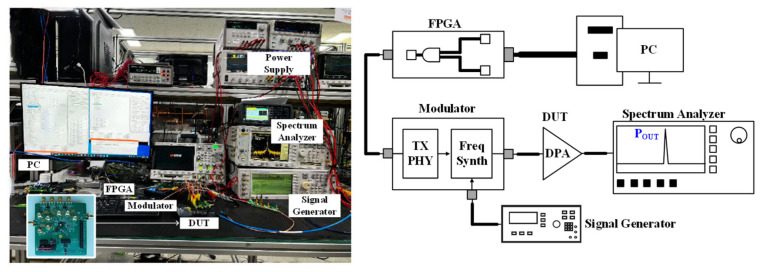
Measurement setup used for the proposed transmitter characterization.

Figure 12 shows the hardware test setup for the fabricated transmitter chip. The chip is embedded on a device under test (DUT) board using a chip-on-board package on a 1.6 mm thick FR4 substrate. The unmodulated and modulated input signals of Gaussian frequency-shift keying (GFSK) and offset quadrature phase-shift keying (O-QPSK) are generated by a PHY transmitter and a modulation device with a two-point modulation synthesizer. The control registers of the DUT and the modulator are configured by a Xilinx Spartan-6 mounted on an integrated FPGA board (Opal Kelly Inc., Portland, OR, USA). The FPGA communicates with the PC via a USB 3.0 module. A signal generator E4433B (Keysight Technologies, Colorado Springs, CO, USA) provides a 10 MHz system clock signal to the DUT and modulator. The output spectrum is analyzed or measured with a spectrum analyzer E4440A (Keysight Technologies, Colorado Springs, CO, USA).

**Figure 13:**
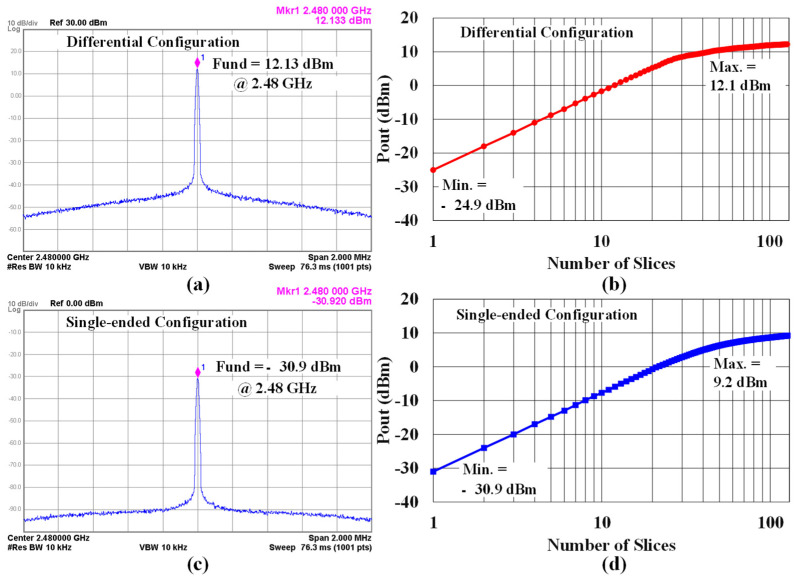
(**a**) The measured maximum output power of the differential CMCD PA, (**b**) its output power as a function of the number of slices, (**c**) the measured minimum output power of the single-ended CMCD PA, and (**d**) its output power as a function of the number of slices.

The maximum output power of the differential CMCD PA is 12.13 dBm for unmodulated data as shown in Figure 13a. In addition, the PA can be digitally tuned between −24.9 and +12.1 dBm by changing the number of slices from 1 to 127 as shown in Figure 13b. The proposed PA also offers a single-ended configuration with a minimum output power of −30.9 dBm for unmodulated data as shown in Figure 13c. Figure 13d shows digitally controllable output powers from −30.9 to 9.2 dBm by varying the number of slices from 1 to 127 for the single-ended configuration. Thus, different output-power levels can be achieved by mixing differential and single-ended configurations.

**Figure 14:**
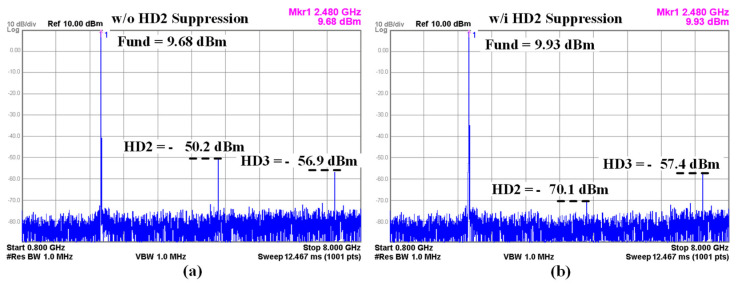
Measured TX typical harmonic: (**a**) without and (**b**) with HD2 suppression.

Figure 14 shows the TX harmonic-suppression performance at the maximum free frequency channel of 2.48 GHz with and without HD2 suppression using duty-cycle distortion correction. Under the +9.68 dBm output-power condition without duty-cycle correction, the harmonic level of HD2 is −50.2 dBm and HD3 exhibits −56.9 dBm as shown in Figure 13a. The harmonic levels can meet the regulatory limit of −41.2 dBm with the help of the HD2 rejection filter and modified C-L-C LPF filter. After enabling duty-cycle correction for HD2 suppression, the fundamental output power is +9.93 dBm, the HD2 level decreases to −70.1 dBm, and the HD3 level achieves −57.4 dBm as shown in Figure 13b. Thus, the HD2 suppression function block is found to improve the HD2 level by 20.2 dB.

**Figure 15:**
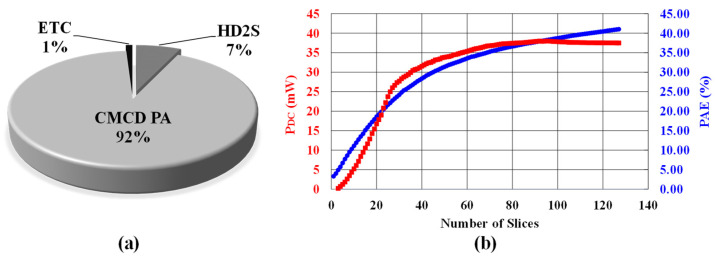
(**a**) Breakdown of measured TX power consumption and (**b**) DC power (P_DC_) and efficiency as a function of the number of slices.

Figure 15a shows the measured power breakdown of the proposed TX. Under the +12.1 dBm output-power condition at 2.48 GHz, the TX consumes 41 mW of DC power (P_DC_). Among the total power consumption, the CMCD PA consumes 92% and the HD2 suppression block consumes only 7% of the total power. Figure 15b shows the DC power (P_DC_) taken from the supply and efficiency that is defined by output power (P_OUT_)/P_DC_ in percentage as a function of the number of slices from 1 to 127. The input signal is a 500-mV peak-to-peak sinusoidal wave. The minimum power consumption is 3.2 mW at −25 dBm P_OUT_ with differential configuration. The maximum efficiency is 40.6% at +12.1 dBm P_OUT_.

**Figure 16:**
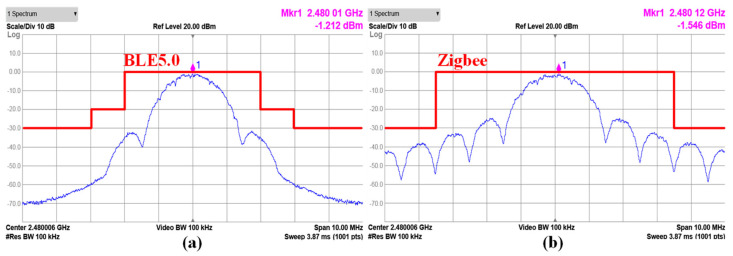
Measured TX spectrum (**a**) at 2 Mb/s for BLE5.0 and (**b**) at 256 kb/s for Zigbee.

Figure 16 shows the TX spectrums of the 2 Mb/s data rate Gaussian frequency-shift keying (GFSK) modulation for the BLE mode and of the 256 kb/s data rate offset quadrature phase shift keying (O-QPSK) modulation for Zigbee mode. The modulated output spectrums are measured with the maximum P_OUT_ setting and a 2^31^ − 1 pseudorandom binary sequence (PRBS). The spectrum for the GFSK modulation has a 14-dB margin from the BLE spectral mask, and the O-QPSK modulation has an 8-dB margin from the Zigbee spectral mask.

The performance of this work is summarized and benchmarked against state-of-the-art TXs operating at the 2.4 GHz ISM band for IoT applications [7,8,10,11,13,14,20,21] in Table 1. The proposed TX achieves the widest *P_OUT_* range. Moreover, HD2 of −82.2 dBc is the lowest level compared to the previous works. The maximum TX efficiency, defined as a percentage by P_OUT_/P_DC_, is 40.6%, and power-added efficiency (PAE), defined as (*P_OUT_ − P_IN_*)/*P_DC_*, is 37.9%. The efficiencies are not the highest rank compared to the previous works due to the many slices of the 7-bit programmable CMCD PA. The transmitter figure-of-merit (TX_FoM) [22] is applied for the fair comparison to account for HD2 rejection performance under limited power consumption and is defined by Equation (4) in dB. The proposed TX ensures the best TX_FoM thanks to the lowest HD2 level by HD2 compression block, HD2 rejection filter, and modified C-L-C filter.

(4) $$HD2+10\log_{10}\left(P_{DC}\right)$$

## 5. Conclusions

The high-efficiency wide-range transmitter using the digitally controlled CMOS current-mode Class-D switching PA is demonstrated in a 28 nm CMOS technology with an area of 0.39 mm^2^. The wide range is achieved by using a mix of differential and single-ended configurations. With the current-domain operation, CMCD PAs can impose zero-voltage switching conditions to mitigate parasitic discharge issues and are suitable for high-power applications with superior efficiency compared to Class E/F PAs. The on-chip XFMR baluns at the output of the PA provide impedance matching along with a low-loss TX switch. The TX maintains sufficiently high HD2 suppression utilizing the proposed duty-cycle distortion correction in addition to the HD2 rejection filter and the modified C-L-C LPF. The proposed TX provides a wide range of power from −31 to 12.1 dBm and achieves a drain efficiency of 40.6% and a power added efficiency (PAE) of 37.9% at maximum output power while consuming 41.1 mW. The suppressed HD2 level is −82.2 dBc at an output power of 9.93 dBm, resulting in an excellent TX_FoM of −97.52 dB. With full on-chip integration and high efficiency, the TX complies with BLE and Zigbee requirements, enabling long-life IoT sensor nodes that ubiquitously support wireless personal area networks (WPANs) and wireless body area networks (WBANs).

## Figures and Tables

**Table 1:** Performance summary of the proposed TX and comparisons to the state-of-the arts.

	This Work	[7] TCAS-I22	[8] ISSCC17	[10] CICC18	[11] TCAS-I18	[13] JSSC15	[14] TCAS-I21	[20] Sensors19	[21] JSSC22 *
Technology (nm)	28	40	55	40	55	55	65	55	28
Supply Voltage (V)	1.0	1.1	1.2	1.1	3	3.3	0.6	3.0	1
PA Topology	Class-D	Class-E	Class-AB	Class-D	Class-D	Class-D	Class-E/F2	Class-D	Class-E
Area (mm^2^)	0.39	0.48	2.2 **	0.7	0.53	N/A	0.85 **	2.9 **	0.112 ***
TX max P_OUT_ (dBm)	12.1	0	8	4	10.05	0	0	0	24.1
TX min P_OUT_ (dBm)	−31	−10	N/A	−10	−23	−20	−6	N/A	5
HD2 @ P_OUT_ (dBc)	−82.2 @ +9.93	−41.3 @ −3	−58 @ +8	−52 @ 0	−45.6 @ +1.6	−54 @ 0	−58 @ 0	−50.07 @ 0	N/A
HD3 @ P_OUT_ (dBc)	−67.3 @ +9.93	−54.2 @ −3	−64 @ +8	−30 @ 0	−50.6 @ +1.6	−52 @ 0	−64 @ 0	−47.08 @ 0	N/A
Max. P_DC_ @ P_OUT_ (mW)	41.1 @ +12.1	4.85 @ 0	79.8 @ +8	N/A	18 @ +10	10.1 @ 0	5.4 @ 0	6 @ 0	N/A
Max. Efficiency @ P_OUT_ (%)	40.6 @ +12.1	20.6 @ 0	7.9 @ +8	N/A	55.6 @ +10	9.9 @ 0	18.5 @ 0	16.7 @ 0	N/A
Max. PAE @ P_OUT_ (%)	37.9 @ +11.9	N/A	N/A	N/A	N/A	N/A	N/A	N/A	50 @ 24.1
P_DC_ @ P_OUT_ (mW)	29.4 @ +9.93	3.22 @ −3	79.8 @ +8	4.5 @ 0	3.9 @ +1.6	10.1 @ 0	5.4 @ 0	6 @ 0	N/A
Efficiency @ P_OUT_ (%)	33 @ +9.93	15.6 @ −3	7.9 @ +8	22.2 @ 0	37.1 @ +1.6	9.9 @ 0	18.5 @ 0	16.7 @ 0	N/A
PAE @ P_OUT_ (%)	32.1 @ +9.93	N/A	N/A	N/A	N/A	N/A	N/A	N/A	N/A
TX_FoM @ P_OUT_ (dB)	−97.52 @ +9.93	−66.52 @ −3	−68.99 @ −3	−75.47 @ 0	−69.69 @ +1.6	−73.96 @ 0	−80.68 @ 0	−72.68 @ 0	N/A

* 2 GHz operation; ** Transceiver (TRX) area; *** only active area.

## Data Availability

Data are contained within the article.
